# Lung B cells in ectopic germinal centers undergo affinity maturation

**DOI:** 10.1073/pnas.2416855122

**Published:** 2025-04-01

**Authors:** Stephane M. Guillaume, William S. Foster, Isabel San Martín Molina, Emily M. Watson, Silvia Innocentin, Grant M. Kennedy, Alice E. Denton, Michelle A. Linterman

**Affiliations:** ^a^Immunology Program, Babraham Institute, Cambridge CB22 3AT, United Kingdom; ^b^Babraham Imaging Core, Babraham Institute, Cambridge CB22 3AT, United Kingdom; ^c^Department of Physics, University of Warwick, Coventry CV4 7AL, United Kingdom; ^d^Department of Immunology and Inflammation, Imperial College London, London W12 0NN, United Kingdom

**Keywords:** tertiary lymphoid structure, germinal center, affinity maturation, iBALT

## Abstract

The lungs are constantly exposed to the external environment and a myriad of antigenic challenges within the air. Chronic exposure to allergens and other airborne antigens can result in the formation of lymphocyte aggregates in the lung, which can harbor ectopic germinal centers (GCs). After allergen exposure, GCs that form in the lung are much smaller and less densely packed with B cells than lymph node GCs. Despite this, ectopic lung GCs support somatic hypermutation and affinity-based maturation as in lymph node GCs, and export memory B cells (MBCs) directly into the lung tissue. This demonstrates that the lung can locally diversify B cell responses and supports the generation of tissue MBC populations in situ.

## Significance

Mature tertiary lymphoid structures (TLSs) in nonlymphoid tissues can sustain germinal center-like structures. This study demonstrates that allergen-induced ectopic germinal centers in the lung, although smaller and slower to respond than those in lymph nodes, are capable of somatic hypermutation and affinity maturation. Notably, high-affinity B cells generated in the lung contribute directly to the local memory B cell pool. These findings underscore the potential of harnessing TLS responses to enhance tissue-resident immunity with the next generation of mucosal vaccines.

Tertiary lymphoid structures (TLSs) are aggregates of lymphocytes that form in nonlymphoid tissues and vary in composition from agglomerations of T cells alone to organized structures that contain functional ectopic germinal centers (GCs) (1). TLSs can contribute to both health and disease depending on the context in which they form. Their presence adjacent to tumors has been associated with better prognosis in multiple cancers (2–5) and improved responses to checkpoint inhibitor therapy (6, 7). TLSs form in the lung following allergen exposure or respiratory infection and often contain ectopic GCs (8–10). Ectopic GCs contribute to disease progression in autoimmune conditions such as rheumatoid arthritis and Hashimoto’s thyroiditis (11, 12), by supporting local antibody responses (13, 14). While these studies show the physiological importance of TLSs in multiple conditions, little is known about their function compared to conventional immune responses in secondary lymphoid tissues.

Secondary lymphoid tissues develop a bespoke stromal cell network that supports and directs lymphocyte responses and GCs during development. However, TLS formation must be preceded by tissue remodeling to develop a fibroblast network that resembles that of secondary lymphoid tissues de novo. For example, induction of the B cell attracting chemokine CXCL13 plays a role in TLS formation, although it is not an absolute requirement (8, 15). This de novo tissue remodeling in response to inflammation results in TLSs, and the GCs within, being structurally different relative to those in secondary lymphoid tissues, likely due to the lack of a preformed stromal cell network (16, 17). Given that GC structure has been linked to the generation of high-affinity antibody responses in secondary lymphoid tissues (18–20), we hypothesized that ectopic GCs are less capable of supporting affinity-based selection, which generates high affinity antibody-secreting plasma cells and memory B cells (MBCs) (21–23).

In conventional secondary lymphoid tissue GCs, GC B cells, T follicular helper (Tfh) cells, T follicular regulatory cells, and macrophages are brought together upon a network of specialized stromal cells including follicular dendritic cells (FDCs) that provide migratory cues that direct cells to, and within, the GC (24). The GC has two functionally distinct compartments known as the light and dark zones. GC B cells localize to the dark zone via expression of CXCR4, which facilitates their migration to the CXCL12-producing reticular stromal cells (18, 25). Here, GC B cells proliferate and their B cell receptor genes undergo somatic hypermutation (SHM). To access positive selection after mutation, dark zone B cells downregulate CXCR4, enabling CXCR5-dependent migration toward the CXCL13-rich FDC stromal network in the light zone. A functional B cell receptor enables them to collect antigen from FDCs, internalize it, and present it to Tfh cells. If the GC B cell can engage a Tfh cell by presenting cognate antigen, it will receive signals to survive and continue maturing. The GC B cells will then either migrate back to the dark zone and undergo further rounds of proliferation and mutation or differentiate into either MBCs or antibody-secreting plasma cells and exit the GC (19, 26–31).

This study characterized ectopic GC formation in the lung tissue after intranasal allergen challenge and assessed the ability of ectopic GCs to generate high-affinity humoral immunity. We found that ectopic GCs are smaller and less densely packed than conventional LN GCs but can support somatic hypermutation and affinity maturation, thus acting as an important local site for high affinity antibody production. The lung was able to contribute to the local MBC pool in response to intranasal challenge. Together this demonstrates that the lung niche can support local high affinity responses without the complex preexisting stromal cell architecture that facilitates a structured GC, as in the lymph node.

## Results

### Intranasal NP-KLH/HDM Immunization Induces Lung and MedLN GC Responses in Parallel.

**Fig. 1.**
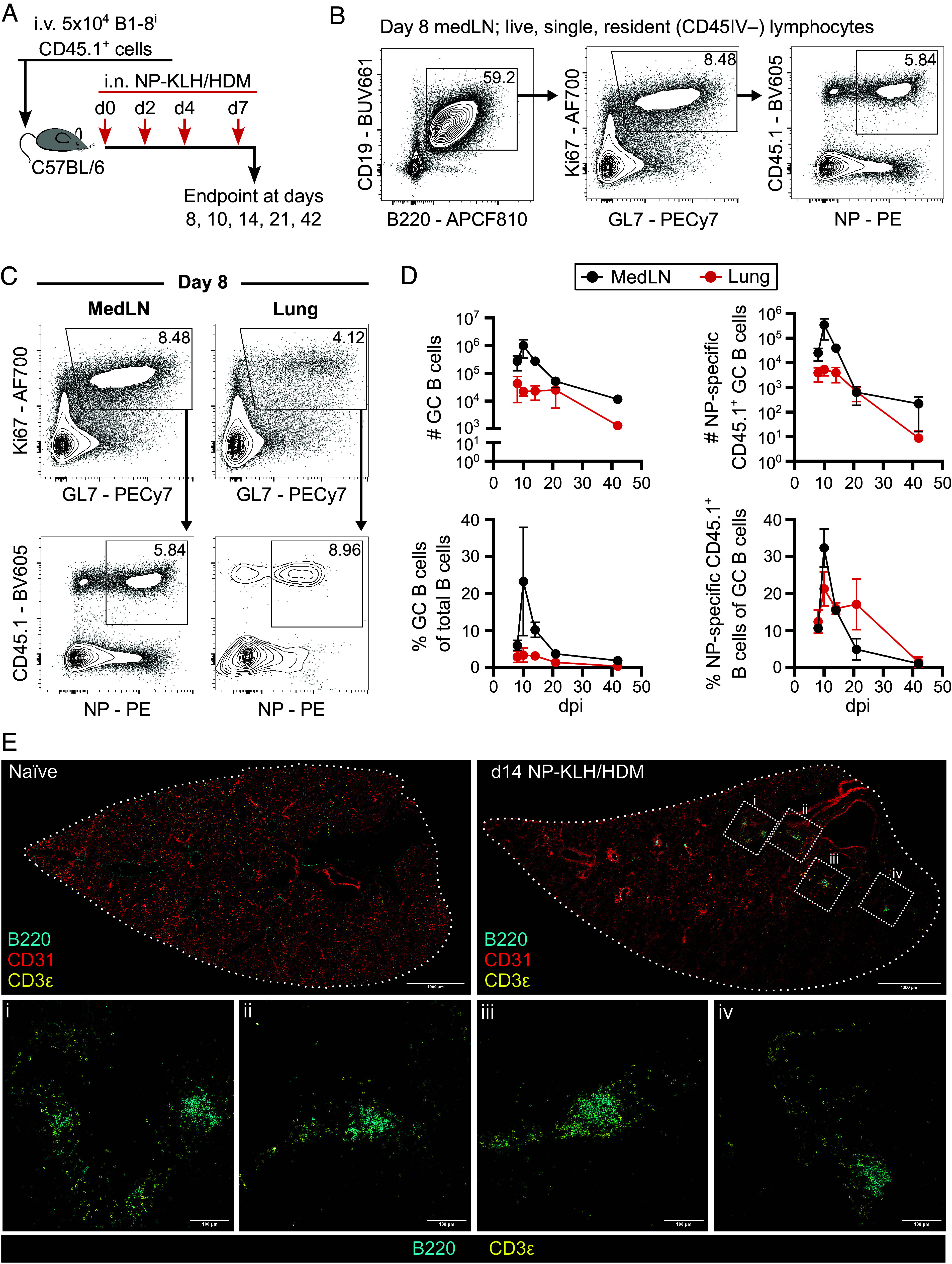
NP-KLH/HDM induces GC formation in the lung and LN in parallel. (*A*) Experimental design of mice receiving adoptive transfer of B1-8^i^ CD45.1^+^ cells, followed by NP-KLH/HDM intranasal immunization. (*B*) Flow cytometric contour plots of population gating strategy, showing B cells (B220^+^CD138^–^CD19^+^CD3^–^), GC B cells (Ki67^+^GL7^+^), and NP-specific cells of interest (NP-bait^+^CD45.1^+^). (*C*) Flow cytometric contour plots depicting GC gating (Ki67^+^GL7^+^) and cell of interest gating (NP-bait^+^CD45.1^+^) in the medLN and lung. (*D*) Quantification and frequencies of Ki67^+^GL7^+^ GC and CD45.1^+^ NP-binding B cells. (*E*) Confocal microscopy of B220 (cyan), CD31 (red), and CD3ε (yellow) staining in lung tissue in naïve mouse (*Upper Left* panel) and d14 after NP-KLH/HDM immunization (*Upper Right* panel). (Scale bar, 1,000 µm.) *Lower* panels show B220 (cyan) and CD3ε (yellow) staining of magnified sections represented by the dashed boxes in d14 NP-KLH/HDM. (Scale bar, 100 µm.) (*B* and *C*) Data are representative of two independent repeats with five mice per timepoint. (*D*) Symbols represent the mean of two experiments, error bars indicate SEM. (*E*) Images are representative of two experiments with two to five mice per group.

To test the hypothesis that ectopic GCs have less affinity maturation, we developed an intranasal dosing system which could allow us to assess the affinity of ectopic lung GCs and directly compare this to conventional LN GCs. We combined the well-used B1-8^i^ 4-hydroxy-3-nitrophenyl acetyl (NP)-specific B cell adoptive transfer model (32) with intranasal allergen challenge. Transfer of B1-8^i^ B cells into C57BL/6 (CD45.2^+^) mice followed by four intranasal doses of NP-keyhole limpet hemocyanin (NP-KLH) mixed with the common allergen house dust mite (HDM) (Fig. 1*A*) induced NP-specific GC B cells 8 d after first dose in both the lung and lung-draining mediastinal lymph node (medLN) (Fig. 1 *B* and *C*). GC B cell and NP-specific CD45.1^+^ numbers and frequencies at 8-, 10-, 14-, 21-, and 42-d postimmunization indicated a similar GC kinetic across tissues, although lung GCs experienced a proportionally lower expansion of B cells compared to lymph node GCs at day 10 (Fig. 1*D*). Immunofluorescent confocal microscopy showed ectopic B cell clusters surrounded by T cells, located adjacent to major vessels and airways in the lung tissue of NP-KLH/HDM treated animals (Fig. 1*E*). This model therefore enables direct comparison of the formation, structure, function, and output of conventional and ectopic GCs.

### Ectopic Lung GCs Are Smaller and Less Densely Packed Than Conventional Lymph Node GCs.

**Fig. 2.**
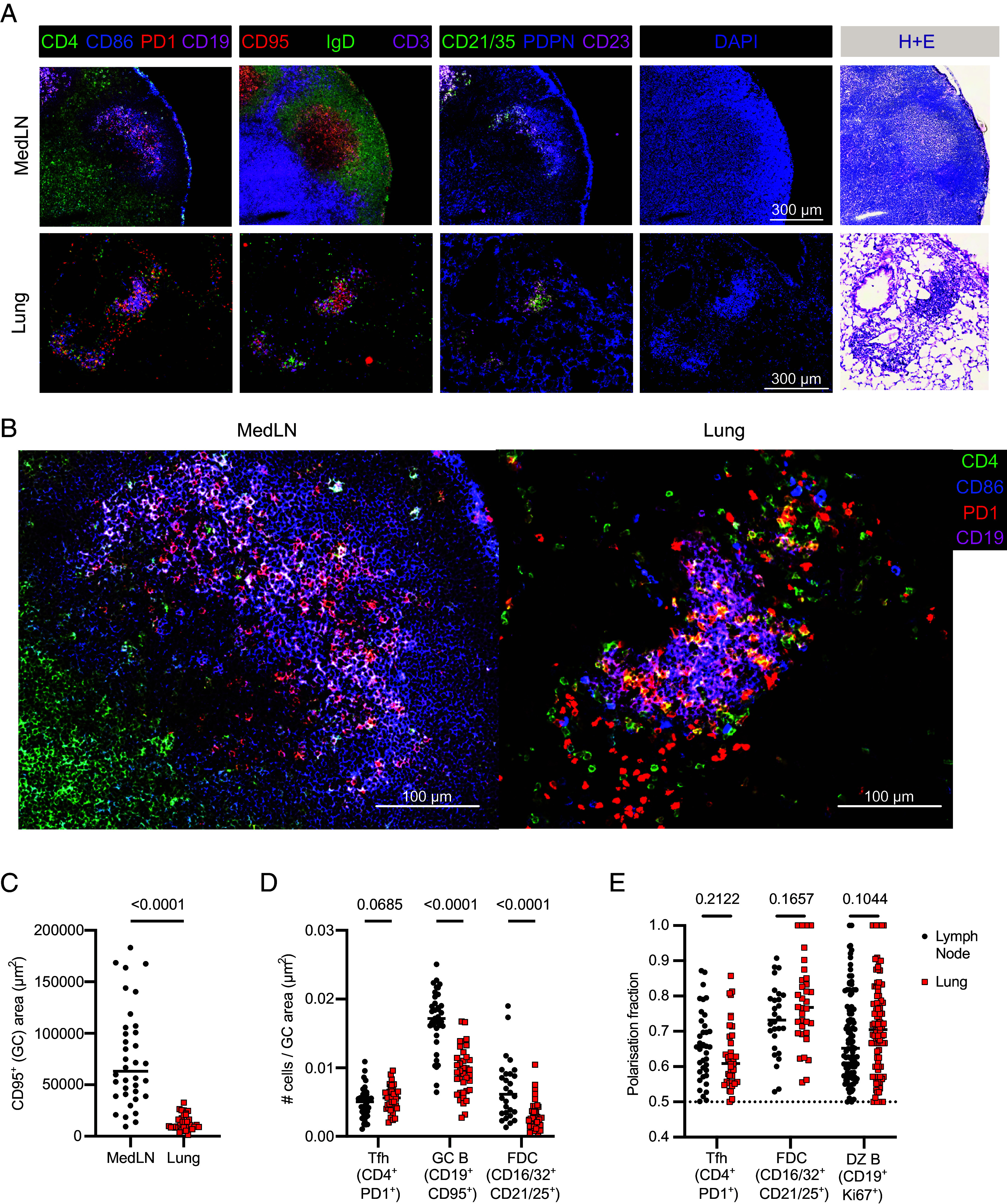
Lymph node GCs are larger than lung GCs with denser GC B cell and FDC populations. (*A*) Representative cyclical staining widefield fluorescence for indicated markers as well as DAPI and Hematoxylin and Eosin stains for medLN and lung GCs. (Scale bar, 300 µm.) (*B*) Zoom images taken from (*A*) (Scale bar, 100 µm.). (*C*) GC area, measured by CD95^+^ staining. (*D*) Cell density for indicated cell types in medLN and lung GCs, calculated as number of cells/GC area. (*E*) Polarization fraction for indicated cell types. (*C*–*E*) Each symbol represents one GC. Two-tailed Mann–Whitney U tests were used. Data are compiled from three sets of MACSima imaging runs and are representative of two independent experiments of five mice each.

To directly compare the structure of lymph node and lung GCs of NP-KLH/HDM-treated mice we used automated cyclic bleaching fluorescence microscopy to identify CD95^+^ GC areas in combination with 23 other parameters, followed by Hematoxylin and Eosin staining of the same tissues (Fig. 2 *A* and *B*). Analysis of the CD95^+^ GC areas showed that ectopic lung GCs were on average 5.7 times smaller than LN GCs (Fig. 2*C*). In both lung and LN GCs, it was possible to identify Tfh cells (CD4^+^PD1^+^), GC B cells (CD19^+^CD95^+^), and FDCs (CD16/32^+^CD21/35^+^). Interestingly, LNs had more GC B cells and FDCs per unit area, suggesting a more tightly packed structure (Fig. 2*D*). Conventional GCs have a compartmentalized structure with polarized light and dark zones, which has been proposed to be missing in ectopic lung GCs (16). We assessed how evenly distributed the Tfh cells and FDC were by computing a GC polarization fraction, in which a value of 0.5 represents an equal distribution of cells across the GC area, and a value of 1.0 represents that all cells compartmentalized in one-half of the GC, for example, solely in the light zone. Although FDCs were less dense in ectopic lung GCs, they were still polarized within the total GC area, as observed in LNs. Consistent with this, Tfh cells and Ki67^+^ B cells were likewise not evenly distributed throughout lung GCs (Fig. 2*E*). Together, this highlights that while ectopic lung GCs are different in both size and density to conventional LN GCs, polarity is still observed in these different cell types.

### Lung and MedLN GCs Both Support Affinity Maturation After NP-KLH/HDM Immunization.

**Fig. 3.**
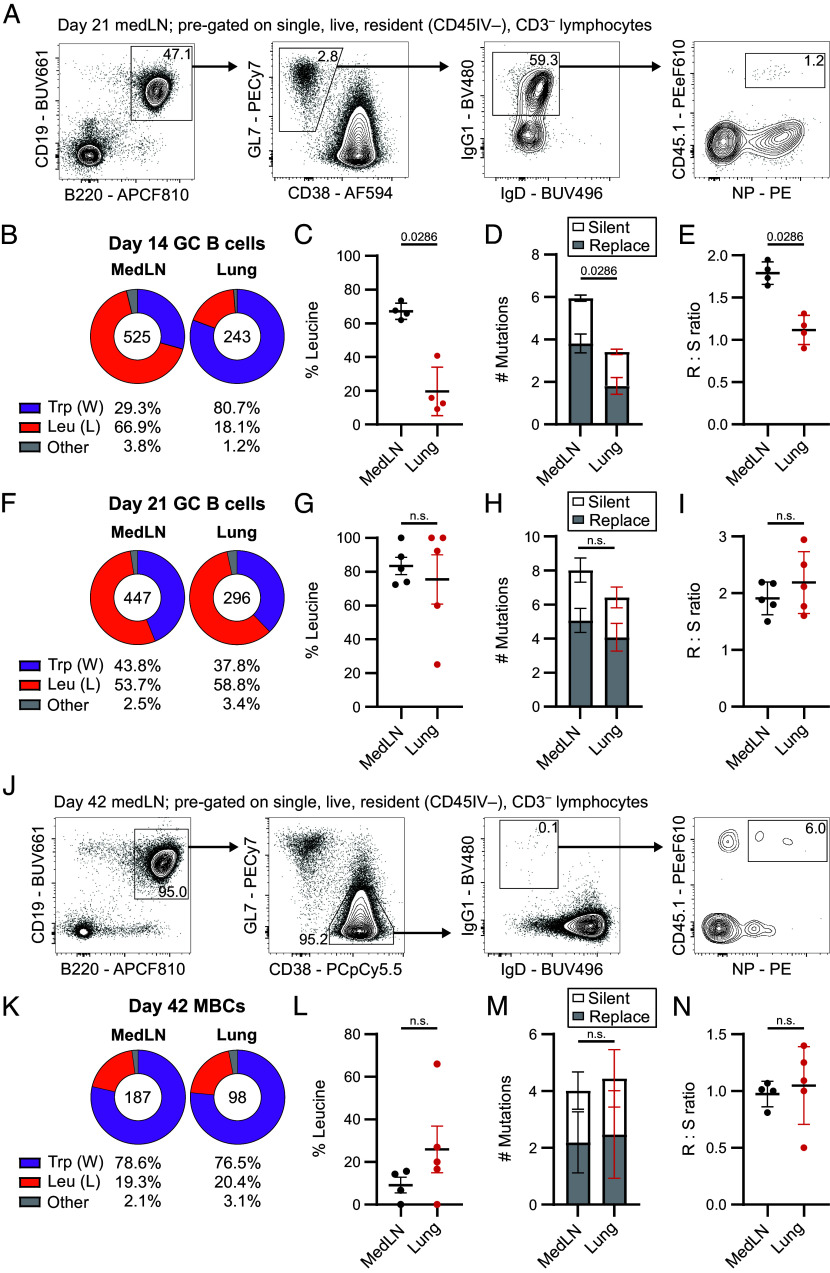
Lung ectopic GCs support similar levels of affinity maturation as mediastinal LN GCs. (*A*) Representative flow cytometry staining of index-sorted lung GC B cells. (*B*) Frequency of cells expressing a tryptophan (“W”; purple), leucine (“L”; orange), or other amino acid (“Other”; gray) at the W33L mutation site of single NP-bait^+^ B1-8^i^ GC B cells sorted at d14. (*C*) Frequency of d14 GC B cells with W33L mutation. (*D*) Silent and replacement mutations in the V_H_186.2 region of d14 GC B cells. (*E*) Replacement to silent mutation ratio in the sequenced V_H_186.2 region of d14 GC B cells. (*F*) Frequency of cells expressing a tryptophan, leucine, or other amino acid at the W33L mutation site of single NP-bait^+^ B1-8^+^ GC B cells sorted at d21. (*G*) Frequency of d21 GC B cells with W33L mutation. (*H*) Silent and replacement mutations in the V_H_186.2 region of d21 GC B cells. (*I*) Replacement to silent mutation ratio in the sequenced V_H_186.2 region of d21 GC B cells. (*J*) Representative flow cytometry staining of index-sorted d42 NP-bait^+^ B1-8^i^ MBCs. (*K*) Frequency of cells expressing a tryptophan, leucine, or other amino acid at the W33L mutation site of MBCs sorted at d42. (*L*) Frequency of d42 MBCs sequenced with W33L mutation. (*M*) Silent and replacement mutations in the V_H_186.2 region of d42 MBCs. (*N*) Replacement to silent mutation ratio in the V_H_186.2 region of isolated d42 MBCs. (*A* and *G*–*I*) Data are representative of three independent experiments with five mice each. (*B*–*E*) Data are representative of two independent experiments consisting of five mice each. (*F*) Data are pooled from three independent experiments consisting of five mice each. Data are from one representative experiment of three independent repeats consisting of five mice each. (*J* and *L*–*N*) Data are representative of two independent experiments with five mice each. (*K*) Data are pooled from two independent experiments consisting of five mice each. (*A* and *J*) Numbers on flow cytometry plots indicate frequency of population gated. (*B*, *F*, and *K*) Numbers inside pie charts indicate number of cells sequenced. (*C*, *E*, *G*, *I*, *L*, and *N*) Symbols represent biological replicates, lines indicate the mean, error bars indicate SD. Mann–Whitney U tests were used. (*D*, *H*, and *M*) Bars indicate the mean, error bars indicate SD. Two-tailed Mann–Whitney U tests were used to compare total number of mutations.

To assess the generation of high affinity B cells within ectopic and conventional GCs, we leveraged the ability of the NP-KLH system to directly assess the frequency of high affinity GC B cells. NP-binding B1-8^i^ cells have tryptophan (W) at the 33rd codon in their V_H_186.2 Cγ1 gene sequence, for which mutation into a leucine (L), “W33L,” confers a ten-fold increase in affinity to the NP hapten (33). Therefore, assessing the proportion of L-bearing CDRs on single B cells provides insight into the acquisition of, and selection for, high affinity mutations in ectopic lung GCs compared to those in the medLN (34). We sequenced the V_H_186.2 Cγ1 region of index-sorted IgG1^+^ NP-specific CD45.1^+^ GC B cells 14 and 21 d after NP-KLH/HDM immunization (Fig. 3*A*). At day 14, 18.1% of lung GC B cells had acquired a leucine compared to 66.9% in the draining lymph node and this was associated with fewer V_H_186.2 Cγ1 mutations and a reduced replacement to silent (R/S) mutation ratio (Fig. 3 *B*–*E*). However, at day 21 a similar proportion of GC B cells in the lung had acquired a leucine (58.8%) as in the medLN (53.7%) (Fig. 3 *F* and *G*). In addition, medLN and lung GC B cells acquired a similar number of total V_H_186.2 Cγ1 mutations and the ratio of R/S mutations across GC B cells was comparable by day 21 (Fig. 3 *H* and *I*). This indicated that affinity maturation can occur in ectopic GCs but is delayed compared to the lymph node.

To investigate whether affinity was preserved in the lung MBC population we index-sorted IgG1^+^ NP-specific MBCs (CD19^+^B220^+^GL7^–^CD38^+^) from the lung and medLN 42 d post immunization (Fig. 3*J*). As anticipated, given their earlier exit from the GC (35), a lower proportion of MBCs contained the high-affinity W33L mutation compared to the GC B cells isolated at day 21 (Fig. 3 *F* and *K*). Like the GC B cell results, lung and medLN MBCs had acquired the W33L mutation in similar frequencies (medLN: 19.3%; lung: 20.4%) (Fig. 3 *K* and *L*), had a similar number of total mutations (Fig. 3*M*) and had no significant difference in R/S mutation ratios (Fig. 3*N*). This evidence suggests that the ectopic lung GC can support similar levels of SHM and selection despite their smaller and less densely packed structure.

To determine whether the intact affinity maturation process in ectopic GCs was unique to our NP-KLH/HDM system, or a broader phenomenon, we compared selection of mutated B cell clones in ectopic lung and conventional LN GCs after influenza A virus infection. We reanalyzed publicly available 10X genomics single-cell RNA sequencing data of mouse IgD^–^ hemagglutinin-specific B cells sorted 7, 14, and 28 d after influenza A infection (36) (*SI Appendix*, Fig. S1*A*). Within the GC B cells, positive selection can be examined via accumulation of replacement mutations in the complementarity determining region (CDR), which can be normalized to the number of silent mutations to generate a metric of selection pressure (Σ) (37), although the selection pressure metric has not been experimentally validated with expressed monoclonal antibodies in previous studies. In this dataset, no lung GC B cells were identified at day 7. We identified a positive selection strength of 0.77 (95% CI: 0.68 to 0.85) in the CDR of medLN GC B cells 14 d postinfluenza A infection, which was significantly greater than the selection strength of 0.09 (95% CI: 0.32 to 0.49) in the lung (*SI Appendix*, Fig. S1 *B*–*D*). Surprisingly, the lung GCs continued to exhibit active CDR selection of 0.32 (95% CI: 0.00 to 0.63) at day 28 that was significantly greater than that observed in the medLN GCs, where the selection strength was −0.27 (95% CI: −0.32 to −0.23, *SI Appendix*, Fig. S1*D*). This indicates that ectopic lung GCs formed in response to viral infection can undergo positive selection in line with affinity maturation, but with slower kinetics compared to conventional LN GCs.

### Lung and Lymph Node GCs Have Limited Clonal Sharing.

**Fig. 4.**
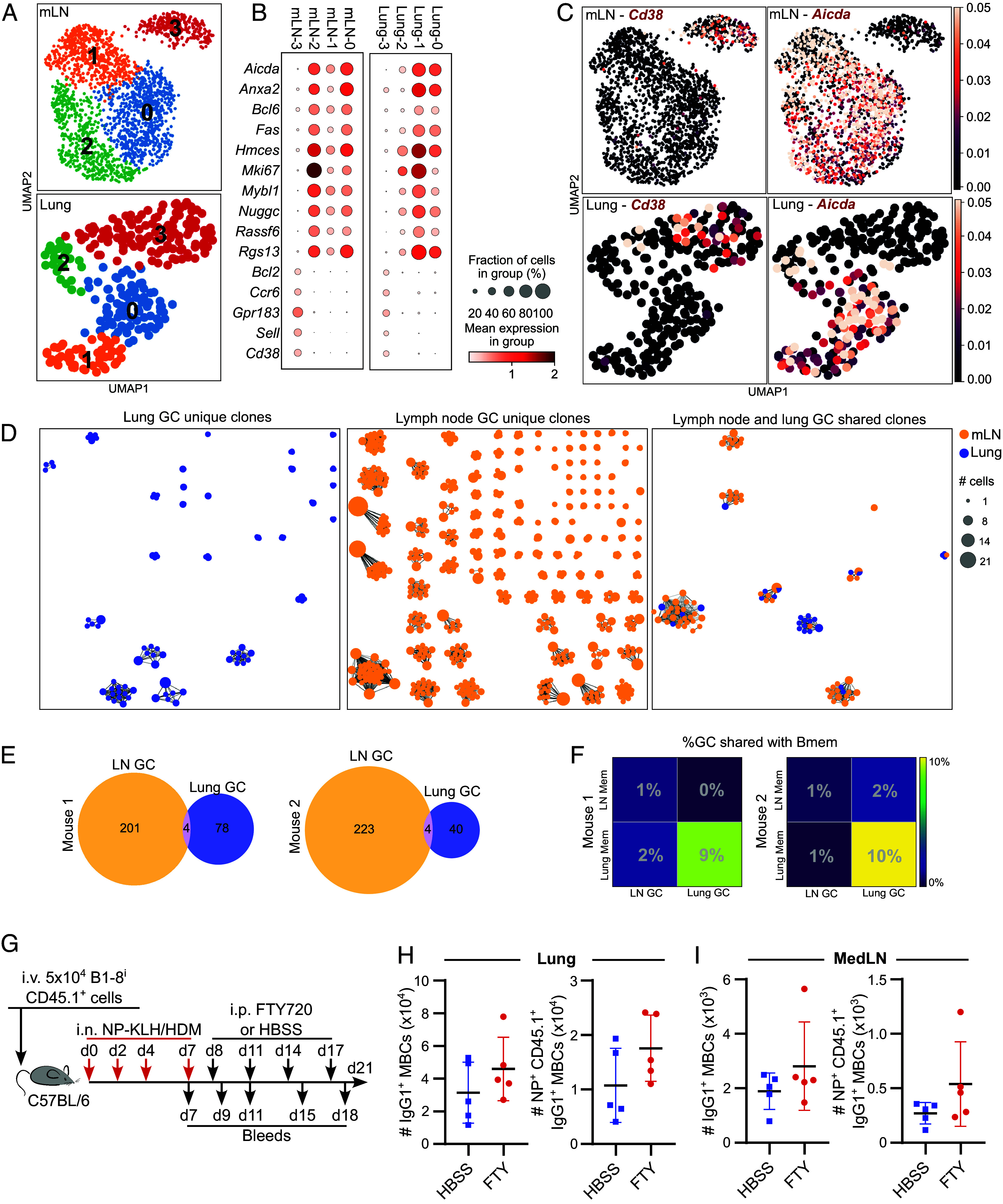
Lung GCs can contribute to the local resident MBC population. (*A*) Leiden clustering of polyclonal GC and MBCs sorted from the mediastinal LN (mLN, *Top* panel) and Lung (*Lower* panel) 21 d after intranasal NP-KLH/HDM as described in Fig. 1. (*B*) Expression of GC B and MBC signature genes in each of the Leiden clusters in (*A*). (*C*) UMAP plots from (*A*) with relative expression of *Cd38* and *Aicda* indicated. (*D*) Stick and ball representation of GC B cell clones in which three or more B cells of the same clone were identified from the lung (blue) and mLN (yellow). Clones only found in the lung GCs are on the *Left*, clones from the LN GCs are in the *Middle*, and shared clones across both tissues are on the *Right*. Data are pooled from two mice. (*E*) Venn diagrams showing the number of shared B cell clones between lung and mLN GCs in two individual mice. (*F*) Heat maps indicating the percentage of GC B cell clones from the lung or mLN that are shared with MBCs from the lung or mLN. (*G*) Experimental design of FTY720 blocking experiment: mice received adoptive transfer of B1-8^i^ CD45.1^+^ cells, followed by NP-KLH/HDM intranasal immunization. Mice received FTY720 or HBSS intraperitoneally every 3 d from day 8 to 17, with regular tail bleeds. (*H* and *I*) Quantification of IgG1^+^ MBCs and NP^+^ CD45.1^+^ IgG1^+^ MBCs in the lung (*H*) and medLN (*I*). Data are representative of two independent repeats with five mice per group. Symbols represent individual mice, lines represent mean, error bars indicate SD. Statistics performed by the two-tailed Mann–Whitney U test.

To assess the clonal relationship between LN and lung GC B cells we performed 10X genomics single-cell BCR and transcriptomics of flow-sorted GC and MBCs from mice 21 d after NP-KLH/HDM administration (38). In both the lung and lymph node samples four clusters were observed (Fig. 4*A*). Cluster 3 had a MBC signature including high expression of *Cd38* which was used to sort MBCs (Fig. 4 *B* and *C*). Clusters 0 to 2 were all enriched for GC B cell genes, including *Aicda,* with a proliferation signature driving the further GC subclustering, indicative of light and dark zone cells (Fig. 4 *B* and *C*), consistent with the polarization of lung GCs observed by imaging (Fig. 2). The BCR sequencing of GC B cells identified clonal expansions (defined as three or more shared GC B cell clones) in both the lung and LN, which were largely, but not exclusively, unique to each tissue (Fig. 4 *D* and *E*). This suggested that the lung and lymph node can support parallel responses, with a small contribution (~2%) of LN derived clones detected in ectopic lung GCs.

### Lungs Are Capable of Locally Generating MBC Populations.

GC-derived MBCs have been proposed to be exported from conventional LN GCs early during the GC response, and shared B cell clones between the medLN and lung have been identified after influenza infection (16, 35). Consistent with this, 21 d after NP-KLH/HDM administration ~1 to 2% of LN GC B cell clones could be found in the lung MBC population. In comparison, 9 to 10% of lung GC B cell clones were represented in the lung MBC pool (Fig. 4*F*). This suggests that both the lung and LN GCs contribute to the lung MBC pool. To formally test whether the ectopic lung GCs can create lung resident memory cells independently of cells that egress from the LN, we administered FTY720 or vehicle control intraperitoneally every 3 d from day 8 until day 17 following B1-8^i^ adoptive cell transfer and NP-KLH/HDM immunization (Fig. 4*G*). FTY720 prevents S1P-mediated egress of lymphocytes from secondary lymphoid organs, thus any lung B cells observed are not from lymph nodes but are locally derived (39). Analysis of blood samples confirmed significantly fewer B and T cells present in the blood following FTY720 treatment (*SI Appendix*, Fig. S2 *A*–*C*). Within the lung, the number of IgG1^+^ MBCs and IgG1^+^ NP-specific CD45.1^+^ non-GC B cells were comparable in the FTY720 and vehicle control groups (Fig. 4 *H* and *I* and *SI Appendix*, Fig. S2 *D* and *E*). These data suggest that pulmonary MBC populations can be seeded by local GCs in response to intranasal NP-KLH/HDM immunization, resulting in a locally diversified population of resident MBCs (40, 41).

## Discussion

The formation of TLSs in nonlymphoid tissues occurs in a myriad of inflammatory conditions including infection, allergy, autoimmune diseases, and cancer (42). These aggregates of lymphocytes, myeloid cells, and stromal cells form de novo in response to chronic inflammation and resemble the immune reactions in secondary lymphoid organs, including the presence of GC-like structures, containing transcriptionally similar cells to GC B cells from lymph nodes (16). Here, we use a modified NP-KLH intranasal immunization protocol, NP-KLH/HDM, to generate GCs both in the LN and ectopically in the lung to facilitate the direct comparison. High parameter immunofluorescence microscopy showed that ectopic GCs are smaller and less densely packed with GC B cells and FDCs than conventional LN GCs. Despite this, the somatic hypermutation and selection process that leads to the accumulation of high-affinity GC B cells is intact in the lung. Further, the lung response was sufficient to seed local populations of MBCs which show hallmarks of GC activity. Together this shows TLSs are capable of locally generating a diversified repertoire of B cells that can provide tissue-specific immunity.

After intranasal NP-KLH/HDM immunization, lung GCs were able to undergo SHM, although the response was delayed compared to the draining lymph node, indicating that these small nonlymphoid tissue GCs are functional. The generation of high-affinity B cells within the lung tissue has the advantage of being able to directly seed the resident MBC pool, which we have shown can come from local GCs, rather than the lung-draining lymph nodes. This corroborates previous research, which has shown that some resident MBCs generated in a particular tissue can be retained locally (35, 43, 44). Because the lung can mount GC responses with functional somatic hypermutation and diversification mechanisms, these ectopic GCs may be important for establishing broadly reactive resident MBC populations in nonlymphoid sites that can help limit reinfections (40, 41). This is particularly relevant as TLSs may also have unique output: B cells found in ectopic GCs formed in response to influenza A infection are more likely to be cross-reactive to multiple influenza strains due to different antigen epitopes being exposed in the lung environment (45, 46). This local generation of immunity could be leveraged via aerosolized vaccines that stimulate lung GC responses that generate MBCs and protect against respiratory infections, provided the vaccines can fully access the lung tissue (47).

In mammalian secondary lymphoid tissues, the structure of the GC has been linked to its function (20, 25, 48). Splenic GCs that exhibit signs of B cell selection and mutation are evolutionarily ancient, as they have been described in nurse sharks, the oldest jawed vertebrate lineage with adaptive immunity (49). Like the ectopic lung GCs observed here, fish and chicken GCs do not show the same organization as GCs in mammalian secondary lymphoid tissues (49–51). These species also tend to develop affinity matured antibody responses at a slower rate than in mammals, as was reported for protective antibodies derived from the mouse lung after influenza A virus infection (9). Together with the work presented here, this indicates that secondary lymphoid tissues likely evolved to support the rapid formation of larger GCs in response to infection, but that ectopic GCs can also perform the same function, albeit with slower kinetics and smaller GCs. This suggests that there may be an evolutionary advantage for retaining ectopic lung GCs in species such as mice and humans, in which lung and LN responses form in parallel. Human infants and young children are at increased risk of viral infections, at least in part due to poor humoral immunity as a result of lower B cell diversity and reduced SHM ability in the early years of life (52, 53). Interestingly, TLSs in pediatric lungs have an increased frequency of GCs that contain B cells specific for respiratory viruses, suggesting that TLSs can provide humoral protection when lymph node responses are less well developed (54). This suggests that vaccines that access the lung may have unique upsides by providing local immune challenge eliciting lung GCs in the early years of life.

## Methods

### Animal Husbandry.

C57BL/6^Babr^ mice and B1-8^i^ mice (32) on the C57BL/6 background were used in this study. Mice were bred and housed in the Babraham Institute Biological Support Unit. Health monitoring reported no additional organisms listed in the FELASA recommendations were detected during surveys of the animal rooms. Rooms are consistently between 19 and 21 °C with a relative humidity of 52%. Lighting was provided on a 12 h light: 12 h dark cycle including 15 min “dawn” and “dusk” periods of subdued lighting. Mice were housed in individually ventilated cages with 1 to 5 mice per cage. Mice were fed CRM (P) VP diet (Special Diet Services) ad libitum and received seeds as part of their environmental enrichment. All mouse experimentation was approved by the Babraham Institute Animal Welfare and Ethical Review Body. Animal husbandry and experimentation complied with existing European Union and United Kingdom Home Office legislation and local standards (PPL: P4D4AF812 and PP9973990). All mice were immunized at between 8 and 16 wk old, both male and female mice were used in the experiments.

### Animal Procedures.

Mice were anaesthetized with inhaled isofluorane, before being immunized with 50 µL PBS containing 100 µg NP-KLH (LGC BioSearch Technologies, Hoddington, UK) and 10 µg HDM (Stallergenes Greer, Baar, Switzerland). At the indicated timepoints post immunization, circulating hematopoietic cells were labeled by intravenous (IV) tail-vein injection of 100 µL PBS containing 5 µg of biotinylated anti-mouse CD45 antibody (BioLegend) 3 min prior to being killed, with lung and draining lymph node taken for analysis. For lymphocyte egress inhibition experiments, FTY720 (Sigma-Aldrich, Gillingham, UK) was reconstituted to a concentration of 2.5 mg/mL in dH_2_O, which was further diluted in HBSS for a working concentration of 0.25 mg/mL. Mice were then administered 100 µL of this solution intraperitoneally (25 µg FTY720 per mouse; approx. 1 µg FTY720 per 1 g mouse).

### Confocal Microscopy.

Lungs were inflated with a 1:1 mix of periodate-lysine-paraformaldehyde (PLP) with optimal cutting temperature compound (OCT) and fixed in PLP for 5 h at 4 °C and then embedded in OCT (55). Lung sections were cut at 30 µm using a CM3050 Cryostat (Leica Biosystems, Wetzlar, Germany), air-dried overnight, and stored at −20 °C. Prior to staining, lung sections were further dried for 1 h at ambient temperature, rehydrated, and blocked with 2% BSA (Sigma-Aldrich) and 10% goat serum (Sigma-Aldrich). Sections were stained with primary antibodies overnight before secondary antibodies were added for 4 h. Images were acquired using a Leica Stellaris 8 microscope. Channels were collected in separate frames using a 20×/0.75 NA air lens. Images were compiled using FIJI (NIH) software (56).

**Table:** 

Target	Clone	Fluorophore	Manufacturer
B220	RA3-6B2	BV605	BioLegend
CD3e	eBio500A2	Purified	ThermoFisher Scientific
CD31	MEC13.3	AF488	ThermoFisher Scientific
αhamster IgG	Polyclonal	AF568	ThermoFisher Scientific
Ki67	Polyclonal	Purified	Abcam
a-rabbit IgG	Polyclonal	AF546	ThermoFisher Scientific

### Flow Cytometry Staining.

Lymph node samples were pressed through a wet 70 µm cell strainer, before being washed through with 3 mL of flow buffer (PBS + 2% FBS + 1 mM EDTA). Lungs were chopped using scissors before being digested for 30 min at 37 °C in a 500 RPM shaker, in RPMI containing 100 µg/mL Liberase TL (Roche, Basel, Switzerland) and 100 µg/mL DNase I (Roche). Lung pieces were then pressed through a wet 70 µm cell strainer. Lung samples were resuspended in ACK red cell lysis buffer, incubated for 5 min and then washed and resuspended in flow buffer. Cell numbers were determined using a CASY TT Cell Counter (OLS OMNI Life Science). Approximately 2 × 10^6^ cells were transferred to V-bottom 96-well plates for staining. Cells were then washed once with FACS buffer, and stained with 100 µL of surface antibody mix (including hemagglutinin B cell probes, streptavidin for CD45-biotin labeling and viability dye) for 1 h at 4 °C. Cells were then washed twice with FACS buffer, and fixed with the eBioscience Foxp3/Transcription Factor Staining Buffer (#00-5323-00) for 20 min at 4 °C. Cells were then washed with permeabilization buffer (eBioscience #00-8333-56) twice and stained with intracellular antibody mix in permeabilization buffer at 4 °C overnight. After 16 h, samples were washed twice with permeabilization buffer and once with flow buffer, resuspended in 200 µL of flow buffer and acquired on a Cytek^TM^ Aurora. Cells for single color controls were prepared in an identical manner to the fully stained samples.

### Antibodies and Proteins Used for Flow Cytometry

**Table:** 

Target	Clone	Fluorophore	Manufacturer
CD45	30-F11	BV510	BioLegend
CD19	1D3	BUV661	BD Biosciences
B220	RA3-6B2	APC-Fire810	BioLegend
BCL6	IG191E/A8	AlexaFluor647	BioLegend
KI67	16A8	AlexaFluor700	BioLegend
IgD	11-26c.2a	SparkNIR685	BioLegend
IgM	II/41	AlexaFluor532	ThermoFisher Scientific
CD38	90	PerCP-Cy5.5	BioLegend
GL7	GL7	PE-Cy7	BioLegend
CD45.1	A20	FITC	BioLegend
CD45.1	A20	BV605	BioLegend
CD45.1	A20	PE-eFluor610	Invitrogen
CD45.2	104	BV510	BioLegend
CD38	90	AlexaFluor594	BioLegend
IgG1	A85-1	BV480	BD Biosciences
IgD	11-26c.2a	BUV496	BD Biosciences
CD45.1	A20	PE-eFluor610	ThermoFisher Scientific
NP-29		PE	LGC Biosearch Technologies
Streptavidin		AlexaFluor350	Invitrogen
Viability dye		Viakrome808	Beckman Coulter
Viability dye		APC-efluor780	ThermoFisher Scientific

### Reanalysis of Bulk BCRseq Data.

Processed 10X sequencing data were sourced from Prof Davide Angeletti (57). Selection strength analysis was performed in R (version 4.3.1) using BASELINe (Bayesian Estimation of Antigen-driven Selection in Ig Sequences), part of the SHazaM package (version 1.2.2) (37, 58).

### Tissue Processing and Cyclic Imaging Using the Automated MACSima Ultrahigh Content Platform in Lung and medLN NP-KLH/HDM Specimens.

14 d postdosing with NP-KLH/HDM, medLNs were processed unfixed, embedded in OCT, and frozen in dry ice at −70 °C until imaging. Lungs NP-KLH/HDM were inflated in PLP as described for confocal imaging. Using a cryostat (CM3050S, Leica), we sectioned a subgroup of medLNs (n = 8) and lungs (n = 4) tissues and collected 8 µm-thick cryosections on SuperFrost Plus adhesion slides (Epredia), air-dried for 30 min at room temperature and inserted into the MACSWell^TM^ One Imaging Frame (Miltenyi Biotec) for imaging with the MACSima. MedLNs were then fixed in 4% formaldehyde buffered solution (pH 6.9, Sigma-Aldrich) for 10 min, and washed in MACSima^TM^ running buffer (Miltenyi Biotec). Both medLNs and lungs were permeabilized with Triton X-100 for 10 min, and prestained with DAPI (1:7) diluted in MACSima^TM^ running buffer to select regions of interest (ROIs). The MACSima platform is an automated imaging system that stains, washes, images, and erases the signal of multiple fluorescent protein markers consecutively in a cyclic manner. The platform is composed of an inverted widefield microscope with a set of LEDs to image DAPI, APC, PE, and FITC fluorescent dyes. The platform also contains a specific bleaching unit for erasing the fluorescent signal in each cycle, which was the method selected for our imaging setup to erase the fluorescent markers in between imaging cycles (59). We used the 2× objective (NA 0.1) for acquiring overview images for ROIs selection based on DAPI staining, then the 20× objective with a NA of 0.75. Highly dynamic range (HDR) photomicrographs were acquired with a monochromatic scientific CMOS (SCMOS) camera, at a pixel resolution of 0.170 µm/pixel. The constant height autofocus method was used for focusing and acquiring the images using the MACSima iQ view software control module (v 1.0.3). 24 antibodies conjugated or targeted with PE and APC fluorochromes were used in the MACSima antibody panel. Average exposure times to acquire the fluorescent signal ranged between 100 and 450%, and the photobleaching values varied between 280 and 600 J and 2 to 6 min. Both fluorescent and background images were acquired per targeted marker in each imaging cycle.

### Antibody Panel Used for Cyclic Imaging at the MACSima Ultraplex Imaging Platform

**Table:** 

Target	Clone	Fluorophore	Dilution	Manufacturer
PD1 (CD279)	RMP1-30	APC	1:50	BioLegend
CD95	REA453	APC	1:30	Miltenyi Biotec
TCRb	REA318	APC	1:50	Miltenyi Biotec
CD3	17A2	AF647	1:50	BioLegend
Podoplanin (gp38)	8.1.1	APC	1:50	BioLegend
CD4	REA604	APC	1:50	Miltenyi Biotec
IgD	REA772	APC	1:50	Miltenyi Biotec
TCR Vδ4	REA372	APC	1:15	Miltenyi Biotec
NK1.1	PK136	APC	1:20	Miltenyi Biotec
IgG1	REA1017	APC	1:50	Miltenyi Biotec
CD8b	REA793	APC	1:50	Miltenyi Biotec
CD185 (CXCR5)	2G8	APC	1:40	BD Biosciences
CD169	REA197	APC	1:50	Miltenyi Biotec
CD138	REA104	APC	1:30	Miltenyi Biotec
CD23	B3B4	PE	1:20	Miltenyi Biotec
CD19	REA749	PE	1:30	Miltenyi Biotec
CD21/35	REA800	PE	1:50	Miltenyi Biotec
CD86 (BT-2)	GL1	PE	1:50	ThermoFisher Scientific
CD16/32	2.4G2	Purified	1:50	BD Biosciences
NP-29		PE	1:150	Biosearch Technologies
Anti-rat IgG (CD16/32)	Secondary antibody (Fcγ fragment)	Cy3	1:50	Jackson ImmunoResearch
CD68	REA835	PE	1:50	Miltenyi Biotec
CD3	REA641	PE	1:40	Miltenyi Biotec
CD27	REA499	PE	1:40	Miltenyi Biotec
CD11b	REA592	PE	1:30	Miltenyi Biotec

### Quantitative Imaging Processing and Analysis of the MACSima Data.

The raw image data obtained from the MACSima platform were processed using the MACSiQ analysis software (v1.3.0). The processing involved flatfield and distortion correction per channel, cycle registration, background subtraction, and camera corrections, as previously described (59). First, each field of view was individually corrected, with 16-bit files being normalized. Exposure times were then adjusted for optimal analysis by correcting overexposed pixels and comparing low and high exposure values for each marker. Camera noise correction was applied by adjusting dark pixels and subtracting camera noise based on calibration values. Next, flatfield correction was applied by inverting and multiplying images and adjusting for the illumination factor. Images were corrected for lens and chromatic distortion, then cropped to the first reference DAPI image and the pixel size was rescaled to 100%. Finally, the processed image was saved in the output folder and opened on the MACSiQ view software for image analysis.

MACSiQ view software was used to extract the number of cells and coordinates of specific clusters of cells, and the area of the GC. To define the GC area, we delineated a ROI based on the expression of CD95 using the tools provided on the MACSiQ view. Within the GC area, the advanced morphology for tissue segmentation algorithm for nuclear segmentation was performed based on DAPI expression. For cytoplasmic segmentation, the constrained donut method was used based on CD3 and IgD expression. Several cell populations including Tfh cells (CD4^+^PD1^+^), GC B cells (CD19^+^CD95^+^), and FDCs (CD16/32^+^CD21/35^+^) were determined using the scatter plots and gating based on cell expression in the workflow editor screen. The GC area, coordinates of each cell population (nucleus X and Y parameters), and the number of cells were extracted from each image.

A “polarization fraction” was computed to define whether these cell type populations were proportionally distributed within the GC area, as follows: the centroids of the GC area and the cells were defined (where the centroid was the average of pixels within the GC area or x and y coordinates of cells). A line was drawn through the GC centroid perpendicular to the line joining the CD95 mask centroid and the cell type centroid, and the number of cells on each side was counted. The polarization fraction was determined by dividing the number of cells on the side with more cells by the total number of cells in the GC (i.e., it is between 0.5 and 1). This fraction was computed independently for each cell type.

### Hematoxylin and Eosin (H&E) Staining and Brightfield Imaging in Lung and medLN NP-KLH/HDM Specimens.

To assess the tissue microarchitecture of both lung and medLN GCs, the Hematoxylin and Eosin stain kit (H&E; VectorLabs) was used post-MACSima. The tissues were hydrated with dH_2_O for 5 min, followed by hematoxylin solution for 3 min, then two quick washes in dH_2_O prior to incubation with bluing reagent (30 s). Sections were then washed twice in dH_2_O and once in 100% ethanol before incubation with eosin solution for 3 min. Finally, sections were dehydrated in 100% ethanol for 3 min and cleared in xylene for 2 min. Histomount medium was used to mount the sections with coverslips (Agar Scientific). Brightfield images were acquired at a resolution of 0.36 µm/pixel using a 20× plan apochromat objective (NA 0.75) and with an exposure time of 3 ms, in the Nikon Ti-2 inverted widefield microscope equipped with a Nikon DS-Ri2 color camera. For visualization of H&E photomicrographs, Nikon Elements software (v 5.41.02) was used to export the images as 8-bit tiff files.

### Nested W33L PCR.

Method adapted from Natt and Espéli (60). In short, cells were index sorted using the FACSAria™ Fusion sorter (BD Biosciences) or FACSDiscover™ S8 Cell Sorter (BD Biosciences) into reverse transcription buffer containing GoScript Reverse Transcriptase (#A5004 Promega), RNase inhibitor (#EO0381 Thermo Fisher Scientific), DTT (#43816 Sigma), Random Hexamers (#SO142 Thermo Fisher Scientific), dNTPs (#R0194 Thermo Fisher Scientific), NP40 (#13021 Sigma-Aldrich), and PBS. Following reverse transcription using a thermocycler (Bio-Rad), samples were subjected to 2 rounds of nested PCR. The PCR mix contained dNTPs (#R0194 ThermoFisher Scientific), HotStar Taq DNA polymerase and PCR buffer (#203205 QIAGEN). For the first round of PCR, cDNA product and the following primers were added to the PCR mix: forward-GCTGTATCATGCTCTTCTTG and reverse-GGATGACTCATCCCAGGGTCACCATGGAGT. The product of the first PCR was then used for the second round of PCR using the PCR mix, along with the following primers: forward-GGTGTCCACTCCCAGGTCCA and reverse-CCAGGGGCCAGTGGATAGAC. Positive clones were identified by running samples on a 1% agarose gel. Positive samples were purified using the ExoSAP-IT PCR Product Cleanup Reagent (#78201 Applied Biosystems) sent for Sanger sequencing to Source Bioscience, UK. Analysis was performed using an automated alignment pipeline (61) that aligned sequences to a V_H_186.2 consensus sequence to identify the 33rd codon for each sample, as well as the quantity of replacement and silent mutations for each sequence.

### Antibodies Used for Fluorescence-Activated Cell Sorting of IgG1+ NP+ B cells, GC B Cells, and MBCs

**Table:** 

Target	Clone	Fluorophore	Manufacturer
CD19	1D3	BUV661	BD Biosciences
Streptavidin		AlexaFluor350	Invitrogen
CD95	Jo2	BUV737	BD Biosciences
IgG1	A85-1	BV480	BD Biosciences
IgG1	A85-1	BV605	BD Biosciences
CD45.2	104	BV605	BioLegend
CD45.2	104	BV510	BioLegend
B220	RA3-6B2	BV785	BioLegend
CD38	90	PerCP-Cy5.5	BioLegend
NP-29		PE	Biosearch Technologies
CD45.1	A20	PE-Cy7	Invitrogen
CD45.1	A20	AlexaFluor700	BioLegend
CD3	17A2	APC	BioLegend
CD4	GK1.5	APC	ThermoFisher Scientific
IgD	11-26c.2a	SparkNIR685	BioLegend
GL7	GL7	PE-Cy7	BioLegend
CD45.1	A20	FITC	BioLegend
CD45.1	A20	APC-eFluor610	Invitrogen
Viability dye		APC-eFluor780	ThermoFisher Scientific

### Single-Cell BCR and RNA Sequencing Sample Preparation.

Following single-cell preparation as described above, single-cell suspensions were washed and resuspended in a fluorescently conjugated antibody cocktail made up in flow buffer and stained for 30 min. Live isotype-switched endogenous germinal center B cells (CD45.2^+^B220^+^CD19^+^IgD^–^CD95^+^CD38^–^) and MBCs (CD45.2^+^B220^+^CD19^+^IgD^–^CD95^–^CD38+) were sorted using a FACSAria Fusion (Becton Dickinson) into flow buffer. Cells were immediately resuspended at 10^6^ cells/mL and processed to gel beads in emulsion (GEMs) using the Chromium iX Controller. Following the manufacturer’s guidelines and input cell stock concentration, the maximum number of cells (18,000) was targeted per sample. Following the GEM generation the library preparation for the whole transcriptome (gene expression) and B cell receptor (BCR) libraries was completed, following the manufacturer’s instructions, using the Chromium GEM-X Single Cell 5′ Kit v3 (PN-1000699/1000695), Chromium GEM-X 5′ Chip Kit (PN-1000698) and Dual Index Kit TT Set A (PN-1000215). Library QC steps were completed using an Agilent 4150 Tapestation with the DNA D1000 High Sensitivity Assay (Agilent, Santa Clara, CA) and qPCR of final libraries with the QuantaBio sparQ Universal Library Quant Kit (QuantaBio, Beverly, MA). Libraries were pooled and sequenced on the AVITI platform using a high output sequencing kit (Element Biosciences, San Diego, CA).

### Single-Cell BCR and RNA Sequencing QC and Analysis.

Single-cell transcriptomics data were processed with CellRanger v5.0 using the mm10-20-A reference genome from 10X. Analysis of transcriptomic data was done in Python using Scanpy (v1.10.4) (62). Cells with <500 or >6,000 genes were discarded, as were cells with >10% mitochondrial genes or 10% in any one gene, and counts for each cell were CLR (centered log ratio) normalized. Cell cycle scores were computed using the list of genes from Tirosh et al. (63). Cell gene expression was reduced to two dimensions with UMAP, and clustered with the Leiden algorithm (64). Cells were classified as GC or memory based on gene expression levels (e.g., *Aicda*, *Cd38*). B cell receptor analysis was performed in Python using Scirpy (v0.20.0) (65), only cells with single V(D)J pairs were kept. Clonotype clusters were found by computing the normalized Hamming distance between junctions for VJ and VDJ sequences and requiring at least 85% junction similarity.

### Statistics.

All statistical tests were run in GraphPad Prism software. Data were assumed to follow non-Gaussian distribution, based on the small sample sized used (n < 10) in each experiment, thus nonparametric statistical tests were used in each analysis. Differences between experimental groups were determined using unpaired two-tailed Mann–Whitney U tests. A threshold of *P* < 0.05 was used to determine statistical significance.

## Supplementary Material

Appendix 01 (PDF)

## Data Availability

Single-cell sequencing data are available at GEO, Accession No. GSE286951 (38). Unique computer code used in the manuscript are available https://github.com/lintermanlab (61). All other data are included in the manuscript and/or *SI Appendix*. Previously published data were used for this work [Data in *SI Appendix*, Fig. S1 were reanalyzed from Mathew et al. (57)].
