# Supplementary material for: Lung B cells in ectopic germinal centers undergo affinity maturation
Source: Proc Natl Acad Sci U S A. 2025 Apr 1;122(14):e2416855122. doi: 10.1073/pnas.2416855122 (PMC12002176; doi:10.1073/pnas.2416855122)
Supplement: Supplementary file 1 — Appendix 01 (PDF) [file pnas.2416855122.sapp.pdf]

Supporting Information for
Lung B cells in ectopic germinal centres undergo affinity maturation.

Stephane M. Guillaume[1, #], William S. Foster[1, #], Isabel San Martín Molina[1,2], Emily M. Watson[1], Silvia Innocentin[1], Grant M. Kennedy[1,3], Alice E. Denton[4, *], Michelle A. Linterman[1, *].

1. Immunology Program, Babraham Institute, Cambridge, CB22 3AT, UK.
2. Babraham Imaging Core, Babraham Institute, Cambridge, CB22 3AT, UK.
3. Department of Physics, University of Warwick, Coventry, CV4 7AL, UK.
4. Department of Immunology and Inflammation, Imperial College London, London, W12 0NN, UK.

[#]Equal contribution, *Equal contribution and Correspondence: Alice Denton a.denton@imperial.ac.uk or Michelle Linterman michelle.linterman@babraham.ac.uk

This PDF file includes:

Figures S1 to S2

Supplementary Figure 1

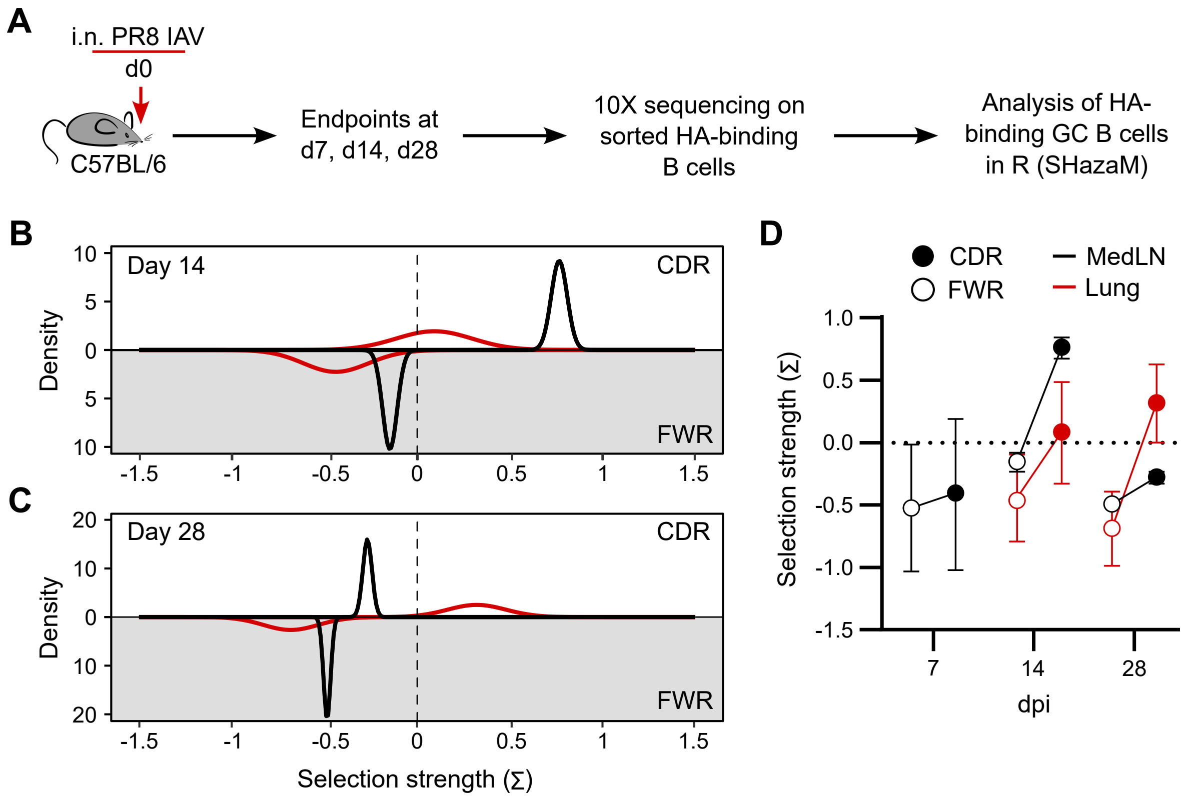

Supplementary Figure 1 – Lung GCs have delayed selection pressure kinetics
(A) Experimental design of PR8 intranasal infection of C57BL/6 mice. **(B, C)** Selection pressure (Σ) analysis of the CDR and FWR of GC B cells isolated from the lung (red) and medLN (black) at day 14 **(B)** and day 28 **(C)**. **(D)** Kinetics of selection pressure in each organ from day 7 to 28; points indicate the mean and error bars indicate the 95% CI. **(B-D)** Data are from one experiment with two to three mice per timepoint.

Supplementary Figure 2

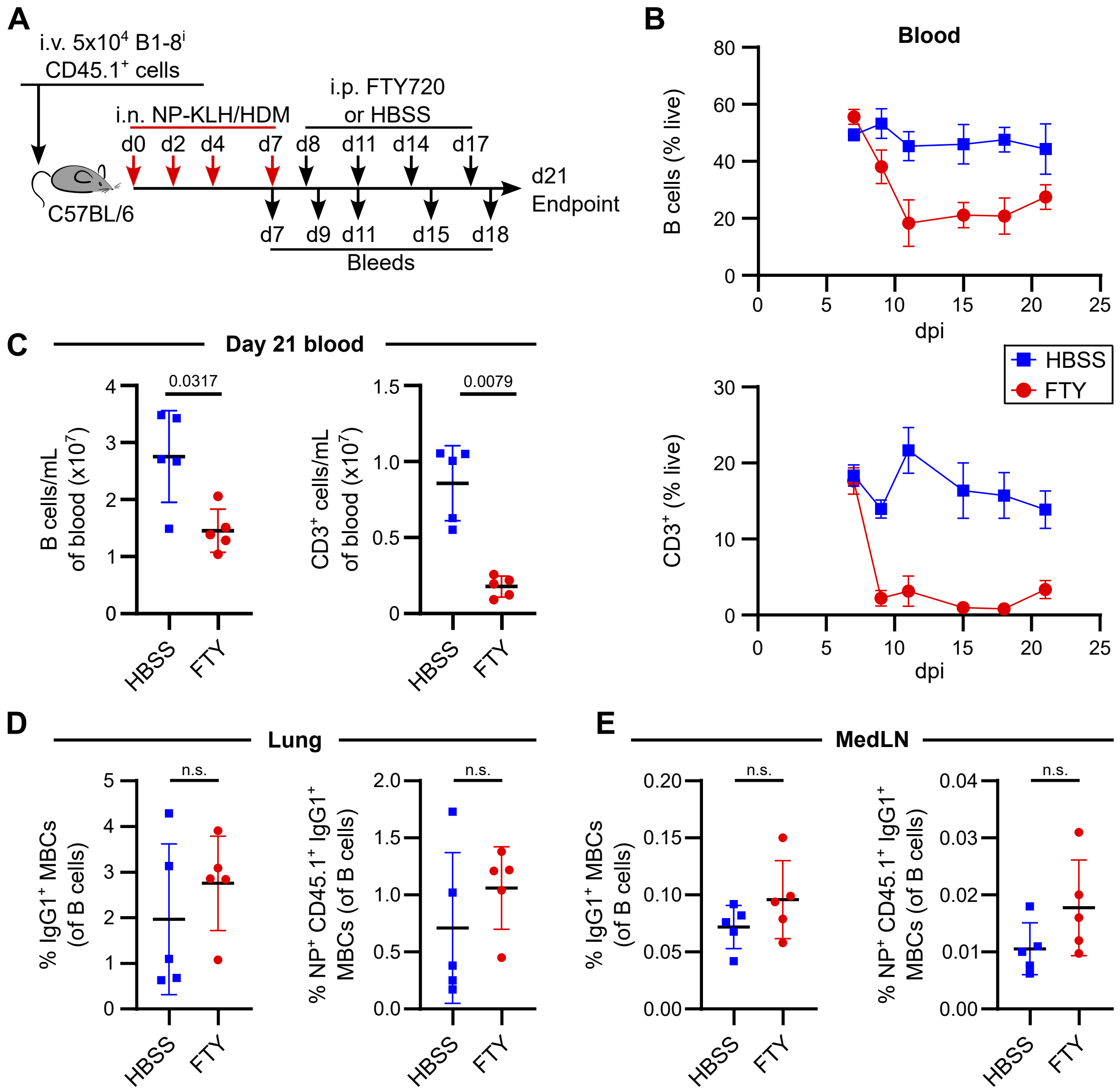

Supplementary figure 2 – Class switched B cells are generated within the lung following NP-KLH/HDM immunisation

(A) Experimental design: mice received adoptive transfer of B1-8^{i} CD45.1^{+} cells, followed by NP-KLH/HDM intranasal immunisation. Mice received FTY720 or HBSS intraperitoneally every three days from day 8 to 17, with regular tail bleeds. **(B)** Cell frequencies in blood samples during HBSS vehicle (blue) or FTY720 (red) treatments. **(C)** Quantification of cells in the blood of mice at d21. **(D, E)** Frequency of IgG1^{+} MBCs and NP^{+} CD45.1^{+} IgG1^{+} MBCs in the lung (D) and medLN (E). **(B-E)** Data are representative of two independent repeats with five mice per group. **(B)** Symbols indicate mean, error bars indicate SD. **(C-E)** Symbols represent individual mice, lines represent mean, error bars indicate SD. Statistics performed by two-tailed Mann-Whitney U test.
